# Airway pressure reliably predicts intra-abdominal pressure during laparoscopic surgery

**DOI:** 10.1007/s10029-026-03763-y

**Published:** 2026-06-26

**Authors:** Pedro Ducatti de Oliveira e Silva, Renato Miranda de Melo, José Fernando Bastos Folgosi, Evandro Rocha Cândido, Roberta Martins Carlos Alves, Ênio Chaves de Oliveira

**Affiliations:** 1 Health Science Postgraduate Programme, Universidade Federal de Goiás (UFG), Goiânia, GO Brazil; 2 Hospital Geral de Goiânia, Goiânia, GO Brasil; 3 Department of Surgery, Faculty of Medicine, Universidade Federal de Goiás, Goiânia, GO Brasil

**Keywords:** Intra-abdominal hypertension, Abdominal compartment syndrome, Airway pressure, Laparoscopic surgery, Intraoperative monitoring

## Abstract

**Background:**

Intra-abdominal hypertension (IAH) and abdominal compartment syndrome (ACS) are life-threatening complications increasingly recognized in the surgical setting, particularly in complex abdominal wall surgery. Bladder pressure monitoring, although the gold standard, is limited intraoperatively due to constraints related to patient positioning, contamination risk, and procedural interruption. We evaluated the correlation between intra-abdominal pressure (IAP) and airway pressure (AWP) during laparoscopic cholecystectomy and the diagnostic accuracy of AWP for detecting intraoperative IAH.

**Methods:**

Prospective observational cross-sectional study in ASA I–II patients undergoing elective laparoscopic cholecystectomy at a single tertiary center, Brazil. Data were collected between 2020 and 2021. Sample size was calculated using the Fleiss method (80% power, two-sided α = 0.05, assumed *r* = 0.30), requiring a minimum of 80 participants. Pneumoperitoneum was increased incrementally (baseline, 5, 10, 15, 20 mmHg). PIP and PLAT were recorded at each level. A linear mixed-effects model (REML) with patient as random intercept was used as the primary correlation analysis; Pearson correlation is reported as a descriptive secondary measure. ROC analyses were performed.

**Results:**

Of 95 patients assessed, 78 completed the study. Both PIP and PLAT correlated significantly with IAP (*p* < 0.001). Linear mixed-effects model: PIP β = 0.439 cmH₂O/mmHg (95% CI: 0.410–0.468), *p* < 0.001, ICC = 0.640. PLAT β = 0.134 cmH₂O/mmHg (95% CI: 0.121–0.147), *p* < 0.001, ICC = 0.936. Pearson r (descriptive): PIP *r* = 0.670 (95% CI: 0.612–0.722); PLAT *r* = 0.253 (95% CI: 0.157–0.343). PIP increased by 6.99 cmH₂O at IAP 15 mmHg and 8.06 cmH₂O at IAP 20 mmHg. ROC analysis showed excellent diagnostic accuracy for PIP (AUC 0.905, 95% CI: 0.868–0.940), with an optimal cutoff of 24 cmH₂O (84.6% sensitivity, 87.2% specificity). PLAT showed poor diagnostic accuracy (AUC 0.695, 95% CI: 0.662–0.737) and is not a reliable clinical surrogate for IAP.

**Conclusion:**

PIP strongly correlates with IAP and accurately detects intraoperative IAH. PLAT is not recommended as a standalone diagnostic surrogate for IAP. Airway pressure monitoring may serve as a practical, real-time screening tool during abdominal surgery, with particular promise in complex abdominal wall reconstruction.

## Introduction

 Intra-abdominal hypertension (IAH) and abdominal compartment syndrome (ACS) are life-threatening complications increasingly recognized in the surgical setting, particularly in complex abdominal wall surgery. The concept of Quaternary Abdominal Compartment Syndrome (QACS), developed specifically for patients undergoing complex ventral hernia repair (VHR), reflects the growing awareness that IAH is not restricted to the critically ill — it represents a relevant intraoperative risk that demands active surveillance [1–4]. Among patients admitted to intensive care units, ACS carries a mortality rate approaching 61%, underscoring the importance of early detection regardless of clinical setting [1].

The gold standard for IAP measurement, as established by the World Society of the Abdominal Compartment Syndrome (WSACS), is bladder pressure monitoring [2, 5]. While well-validated in the intensive care unit, this method carries significant practical limitations in the operating room. Surgical table repositioning continuously alters the pressure reference point; catheter manipulation under the surgical field raises contamination risk; and each measurement requires interrupting the operative procedure [6]. These constraints make continuous intraoperative IAP surveillance through bladder pressure monitoring impractical, creating a critical monitoring gap during the very procedures in which IAH is most likely to develop [7, 8].

Airway pressure (AWP) has been proposed as a practical surrogate for IAP, grounded in the anatomical and physiological integration of the thoracic and abdominal compartments through the diaphragm [9–13]. Peak inspiratory pressure (PIP) and plateau pressure (PLAT) are continuously displayed on intraoperative monitors, requiring no additional equipment or procedural interruption. Surgeons and anesthesiologists worldwide already use AWP changes informally to suspect IAH — yet this clinical practice lacks a rigorous human evidence base [14, 15]. The only studies establishing IAP–AWP correlation to date were conducted in porcine models, without incremental IAP titration, and their authors explicitly called for validation in humans [16, 17].

Laparoscopic cholecystectomy offers a uniquely controlled human model for this investigation. Performed under general anesthesia with standardized ventilatory parameters, it allows direct, continuous IAP measurement through the insufflator while simultaneously recording AWP — with confounding variables held stable. The aims of this study were therefore to evaluate the correlation between AWP (PIP and PLAT) and IAP across incrementally titrated pneumoperitoneum levels, and to determine the diagnostic accuracy of AWP for detecting intraoperative IAH.

## Methods

### Study design and setting

This prospective, observational, cross-sectional study assessed airway pressures during pneumoperitoneum installation in patients undergoing elective laparoscopic cholecystectomy at Hospital Geral de Goiânia (Goiânia - GO, Brazil), a tertiary public hospital. Data were collected between January 2020 and December 2021. The study was approved by the Hospital Research Ethics Committee (CAAE No. 33840120.4.0000.0035), and all participants provided written informed consent. To minimize measurement bias, a standardized ventilatory and anesthetic protocol was maintained throughout data collection, including continuous TOF and BIS monitoring. No sensitivity analyses were prespecified.

### Sample size

Sample size was calculated using the Fleiss method, with 80% statistical power, two-sided significance level of 5%, and an assumed Pearson correlation coefficient of *r* = 0.30 between IAP and PIP based on porcine model data [16, 17], yielding a minimum required sample of 80 participants.

### Patient selection

**Fig. 1:**
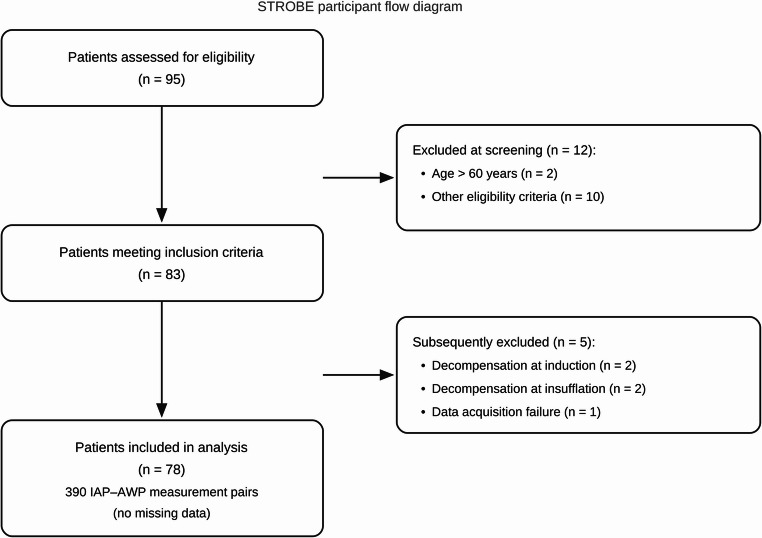
STROBE participant flow diagram

Patients with symptomatic cholelithiasis of both sexes, aged up to 60 years, classified as ASA physical status I or II, and without underlying pulmonary disease were eligible. Consecutive patients were invited during the pre-anesthetic consultation. Patients who experienced clinical decompensation during anesthetic induction or pneumoperitoneum insufflation, or in whom data acquisition was incomplete, were excluded. The flow of participants is presented in Fig. 1.

### Anesthetic and ventilatory protocol

All patients underwent balanced general anesthesia. During anesthetic induction and the pneumoperitoneum titration phase, patients were maintained in the supine neutral position; reverse Trendelenburg positioning was applied only after completion of the data collection phase, when laparoscopic cholecystectomy proceeded in the standard fashion. This standardization ensured that positional changes did not confound airway pressure measurements. Mechanical ventilation was delivered in volume-controlled mode using a lung-protective strategy: tidal volume of 6 mL/kg predicted body weight (Devine formula: height [cm] − 100 for men; height [cm] − 105 for women), respiratory rate of 12–16 breaths per minute, and PEEP ≤ 5 cmH₂O. Neuromuscular blockade was monitored by train-of-four (TOF) stimulation— airway pressures were recorded only when TOF ratio ≥ 0.9, confirming full neuromuscular blockade at each measurement point. Anesthetic depth was assessed using the Bispectral Index (BIS), maintained between 40 and 60.

### Airway pressure measurements

Airway pressures were recorded from a multiparametric anesthesia monitor (WATO^®^ EX-65, Mindray). Two parameters were analyzed: peak inspiratory pressure (PIP) and plateau pressure (PLAT), both obtained in cmH₂O (conversion factor: ×0.74 = mmHg).

### Pneumoperitoneum protocol and data collection

Pneumoperitoneum was established using an endoscopic insufflator (Endoflator^®^ 40, Karl Storz) with CO₂ at 3 L/min. IAP was measured via the insufflator readout display, which provides continuous real-time pressure values. IAP was increased incrementally: baseline (IAP = 0 mmHg, prior to insufflation), 5, 10, 15, and 20 mmHg. At each level, a one-minute stabilization period was observed before recording. Data were complete for all 78 patients across all five measurement points (390 IAP–AWP pairs; zero missing values).

### Statistical analysis

The primary analysis of the association between IAP and AWP was performed using a linear mixed-effects model (LME) with restricted maximum likelihood (REML) estimation, including IAP as a fixed effect and patient as a random intercept, to account for within-patient dependency across repeated measurements. The intraclass correlation coefficient (ICC) was calculated to quantify the proportion of total variance attributable to between-patient differences. The Pearson correlation coefficient with 95% confidence intervals (Fisher’s z-transformation) is reported as a descriptive secondary measure. Group differences across IAP levels were assessed by one-way ANOVA with Tukey’s post hoc test. Diagnostic accuracy of PIP and PLAT for detecting IAH (IAP ≥ 15 mmHg per WSACS criteria [2]) was assessed by ROC curve analysis; optimal cutoff by Youden’s index. Bootstrap 95% confidence intervals (2,000 resamples) were calculated for all AUC estimates. BMI-stratified ROC analyses were performed as pre-specified subgroup analyses. Exploratory factorial ANOVA evaluated IAP–PIP interactions by BMI, smoking, sex, age group, and opioid type. Significance level: 5%. IBM SPSS Statistics version 26.0.

## Results

### Patient flow and baseline characteristics

Initially, 95 patients were assessed, 83 met inclusion criteria; 12 were excluded at screening (age > 60 years: *n* = 2; other eligibility criteria: *n* = 10). Of 83 eligible patients, 5 were subsequently excluded (decompensation at induction: *n* = 2; decompensation at insufflation: *n* = 2; data acquisition failure: *n* = 1), resulting in 78 patients for analysis (Fig. 1). The sample was predominantly female (76.9%), mean age 38.4 ± 12.5 years, mean BMI 27.9 ± 5.4 kg/m². Mean baseline PIP was 18.1 ± 3.4 cmH₂O and PLAT 8.3 ± 2.9 cmH₂O (Table 1).

**Table 1:** Baseline characteristics of the study population (*n* = 78)

Variable	Value
Age, mean ± SD (years)	38.4 ± 12.5
Sex, female, n (%)	60 (76.9%)
Weight, mean ± SD (kg)	74.5 ± 17.7
Height, mean ± SD (m)	1.63 ± 0.10
BMI, mean ± SD (kg/m²)	27.9 ± 5.4
BMI ≥ 25 kg/m², n (%)	52 (66.7%)
ASA I, n (%)	66 (84.6%)
ASA II, n (%)	12 (15.4%)
Never smoked, n (%)	54 (69.2%)
Hypertension, n (%)	11 (14.1%)
Diabetes mellitus, n (%)	2 (2.6%)
Baseline PIP, mean ± SD (cmH₂O)	18.1 ± 3.4
Baseline PLAT, mean ± SD (cmH₂O)	8.3 ± 2.9

*SD* standard deviation; *BMI* body mass index; *ASA* American Society of Anesthesiologists; *PIP* peak inspiratory pressure; *PLAT* plateau pressure. No missing values

### Correlation between IAP and airway pressures

**Fig. 2:**
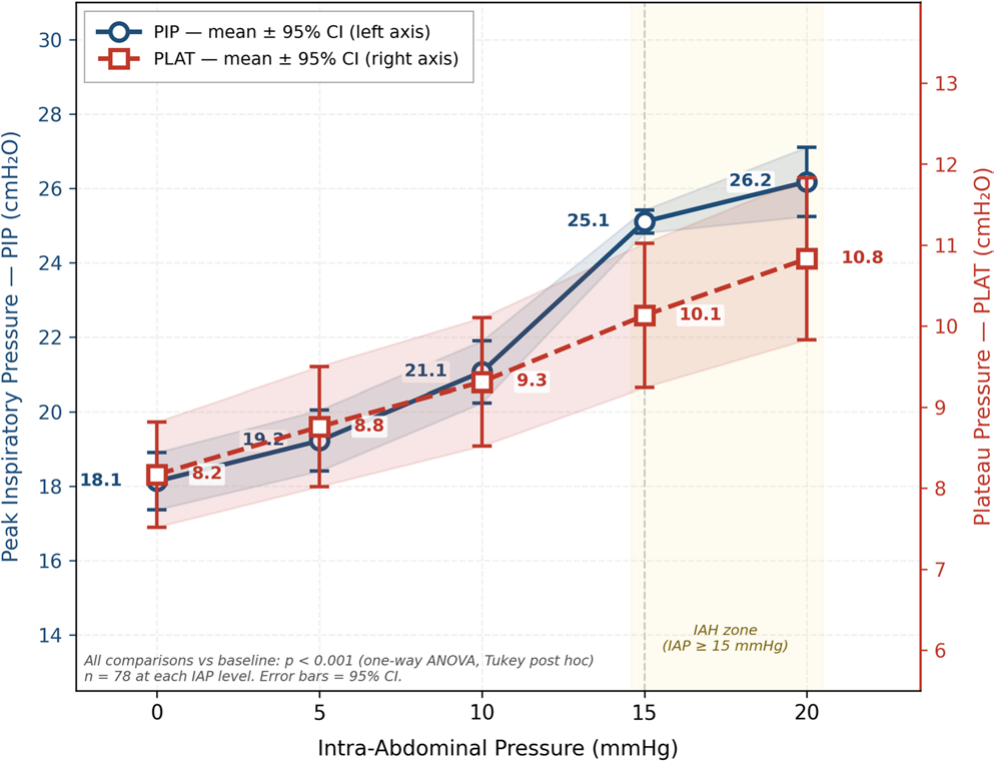
Peak inspiratory pressure (PIP) and plateau pressure (PLAT) across intra-abdominal pressure (IAP) levels

Linear mixed-effects model (REML): IAP was a significant fixed-effect predictor of PIP (β = 0.439 cmH₂O per mmHg; 95% CI: 0.410–0.468; z = 29.61; *p* < 0.001) and of PLAT (β = 0.134 cmH₂O per mmHg; 95% CI: 0.121–0.147; z = 20.21; *p* < 0.001). The ICC was 0.640 for PIP and 0.936 for PLAT, indicating that 64% of PIP variability and 93.6% of PLAT variability are attributable to between-patient differences in baseline values. As a descriptive secondary measure, Pearson correlation confirmed a moderate positive association for PIP (*r* = 0.670, 95% CI: 0.612–0.722, *p* < 0.001) and a weak association for PLAT (*r* = 0.253, 95% CI: 0.157–0.343, *p* < 0.001). Mean PIP and PLAT values at each IAP level are presented in Fig. 2; Table 2.

**Table 2:** Mean airway pressure values at each IAP level

IAP (mmHg)	PIP mean ± SD (cmH₂O)	PLAT mean ± SD (cmH₂O)
0 (baseline)	18.1 ± 3.4	8.3 ± 2.9
5	19.2 ± 3.7	8.8 ± 3.3
10	21.1 ± 3.7	9.5 ± 3.7
15	25.1 ± 1.4	10.3 ± 4.1
20	26.2 ± 4.1	11.0 ± 4.6

*n* = 78 at each level; zero missing values

Post-hoc analysis (Tukey) confirmed baseline PIP differed significantly from all subsequent IAP levels (all *p* < 0.001), as did baseline PLAT (all *p* < 0.001). PIP increased by 6.99 cmH₂O (± 3.15) at IAP 15 mmHg and 8.06 cmH₂O (± 2.66) at IAP 20 mmHg. PLAT increased by 2.02 cmH₂O (± 1.78) and 2.75 cmH₂O (± 2.31) respectively. The difference in PIP between IAP 15 and 20 mmHg was numerically small (mean 1.07 cmH₂O), suggesting a plateau in PIP response at higher IAP levels.

### Diagnostic accuracy of airway pressure for IAH detection

**Fig. 3:**
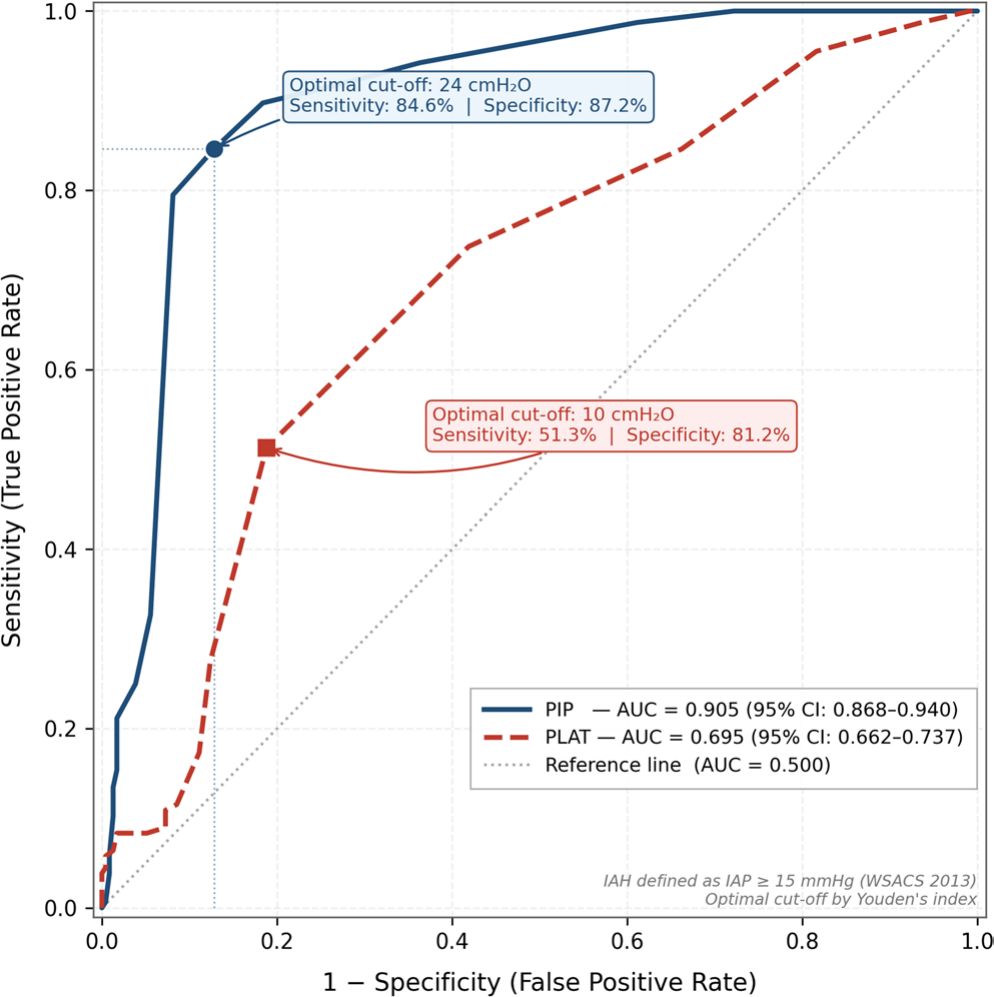
Receiver operating characteristic (ROC) curves for PIP and PLAT in the detection of intra-abdominal hypertension

ROC analysis for PIP in relation to IAP ≥ 15 mmHg demonstrated excellent diagnostic accuracy: AUC = 0.905 (95% CI: 0.868–0.940). Using Youden’s index, the optimal PIP cutoff was 24 cmH₂O (sensitivity 84.6%, specificity 87.2%). PLAT showed substantially lower diagnostic accuracy (AUC = 0.695, 95% CI: 0.662–0.737; optimal cutoff 10 cmH₂O, sensitivity 51.3%, specificity 81.2%) and is not considered a reliable surrogate for IAP detection (Fig. 3). The positive likelihood ratio for PIP was 19.3 and the negative likelihood ratio 0.04.

### Subgroup analyses

**Fig. 4:**
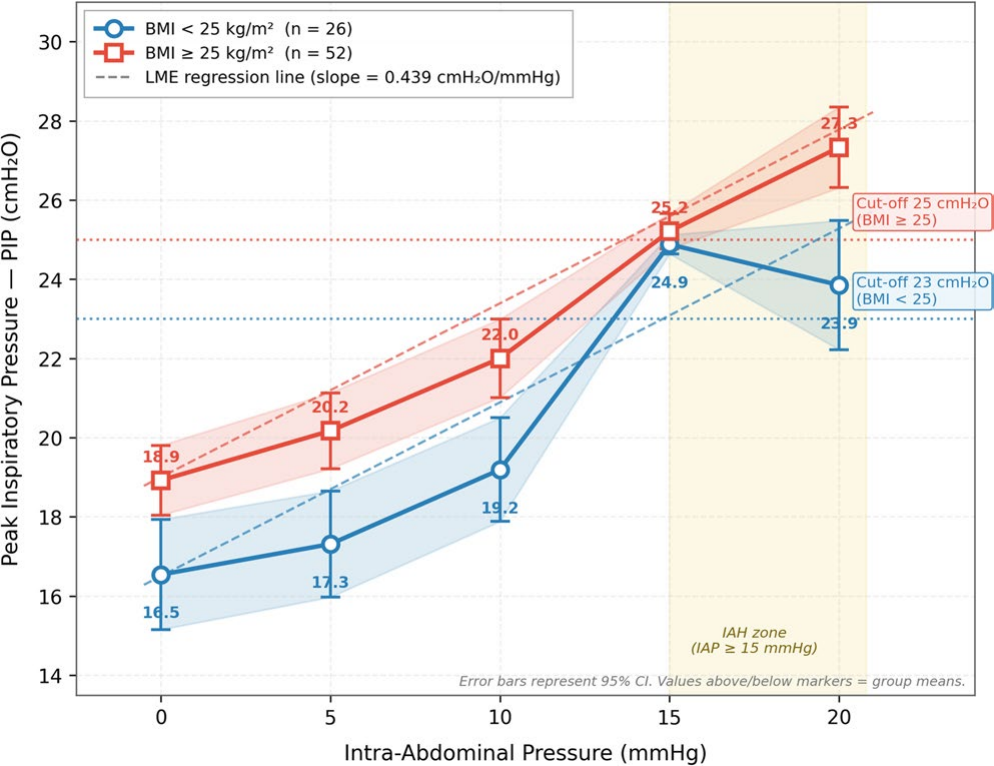
BMI-stratified relationship between PIP and IAP

Factorial ANOVA confirmed consistent IAP–PIP association across age groups (interaction *p* = 0.957) and sexes (interaction *p* = 0.993). BMI significantly influenced baseline PIP (16.5 ± 3.4 cmH₂O in BMI < 25 vs. 19.0 ± 3.2 cmH₂O in BMI ≥ 25; *p* < 0.001). BMI-stratified ROC analysis (pre-specified) identified: BMI < 25 kg/m² — optimal cutoff 23 cmH₂O (AUC 0.920, 95% CI 0.869–0.963; sensitivity 80.8%, specificity 91.0%); BMI ≥ 25 kg/m² — optimal cutoff 25 cmH₂O (AUC 0.908, 95% CI 0.857–0.956; sensitivity 83.7%, specificity 89.7%). Mixed-effects interaction analysis confirmed that the difference in cutoffs reflects higher baseline PIP in overweight patients (β = +2.442 cmH₂O, *p* = 0.001) rather than a different PIP response slope to IAP elevation (interaction *p* = 0.826). The BMI-stratified interaction plot is shown in Fig. 4. Active smokers showed an attenuated PIP response at IAP > 15 mmHg; however, given the very small subgroup size (*n* = 3), this observation should be considered hypothesis-generating only and is not suitable for clinical inference. Remifentanil was associated with higher absolute PIP values across all IAP levels, consistent with its thoracic rigidity profile.

## Discussion

This prospective study demonstrates that peak inspiratory pressure correlates significantly with incremental increases in IAP during laparoscopic surgery, and that PIP provides excellent diagnostic accuracy for identifying IAH. The primary analysis using a linear mixed-effects model, which appropriately accounts for the repeated-measures structure of the data, confirms a robust fixed-effect slope of 0.439 cmH₂O per mmHg of IAP (ICC = 0.640), supporting the validity of the correlation independent of between-patient variability. These findings support the use of airway pressure monitoring as a practical intraoperative surrogate for IAP.

There is strong physiological plausibility in the relationship between intra-abdominal and airway pressures [18]. Since the patient is connected to the ventilator during general anesthesia, monitoring PIP is immediately attractive: it is displayed automatically and in real-time, requiring no additional equipment or procedural interruption. Two previous studies with similar methodology observed this correlation in porcine models but did not titrate IAP incrementally: O’Mara et al. [16] and Mohan et al. [17]. The present study is the first to establish this correlation in humans with incremental titration. Of note, the sample size was calculated a priori assuming a conservative correlation of *r* = 0.30 derived from these porcine models [16, 17]; the correlation subsequently observed between PIP and IAP in humans (*r* = 0.670) was more than double the assumed value. This indicates that the study was conservatively powered and that the human IAP–PIP association is considerably stronger than the animal-model estimate on which the design was based.

The abdominal compliance response to increasing abdominal volume follows three theoretical phases: reshaping, stretching, and pressurization [19]. The PIP curve observed in Fig. 2 mirrors this behavior, with a steeper rise at higher IAP levels consistent with reduced abdominal wall compliance. The small difference in PIP between IAP levels of 15 and 20 mmHg likely reflects attenuated abdominal wall compliance at high pressures, supporting a binary screening role for PIP — alerting the team when PIP crosses the identified cutoff — rather than a role in tracking each incremental IAP change.

The high ICC of PLAT (0.936) provides a mechanistic explanation for its poor diagnostic accuracy: 93.6% of PLAT variability is determined by fixed patient-level characteristics — particularly static chest wall compliance — rather than by dynamic IAP changes. It is acknowledged that compliance-derived indices (e.g., dynamic compliance = tidal volume / [PIP − PEEP]) would theoretically remove the confounding effect of airway resistance. However, PIP is preferred in the clinical context because it is continuously displayed on every modern anesthesia monitor without requiring additional calculation. PLAT is not recommended as a standalone clinical surrogate for intraoperative IAP.

Our results corroborate findings from a Portuguese retrospective study assessing QACS in complex VHR [20]. Chandra et al. reported a PIP threshold of approximately 30 cmH₂O for identifying patients requiring prolonged intubation [21], higher than our cutoff, consistent with higher baseline PIP in that population. Blatnik et al. observed that both PIP and PLAT changes predicted respiratory complications after abdominal wall reconstruction [15]. A separate cohort showed a mean PLAT change of 5.4 cmH₂O [22]; differences from our findings are likely explained by patient characteristics, particularly age, BMI, and baseline IAP.

Subgroup analysis revealed that patients with BMI ≥ 25 kg/m² have higher baseline PIP but an identical response slope to IAP elevation — a finding that directly informs the BMI-stratified cutoffs identified (23 cmH₂O for BMI < 25; 25 cmH₂O for BMI ≥ 25).

A secondary observation that merits comment is the association between remifentanil and higher absolute PIP values across all IAP levels. Potent synthetic opioids—including remifentanil and fentanyl—can produce skeletal muscle rigidity of the thoracic and abdominal wall, classically termed opioid-induced (“wooden chest”) rigidity, which reduces chest wall compliance and can raise peak airway pressure independently of intra-abdominal pressure. This effect is most pronounced after rapid, high-dose bolus administration and is largely attenuated by neuromuscular blockade, which was maintained throughout our measurements (TOF ratio ≥ 0.9); the residual association observed here should therefore be interpreted as hypothesis-generating rather than definitive. Importantly, remifentanil appeared to shift the absolute PIP value rather than the slope of the PIP–IAP relationship, so the role of PIP as a within-patient trend from baseline is preserved. Nevertheless, because opioid choice may shift baseline airway pressure, opioid regimen is a variable that warrants control in future studies and further supports interpreting PIP relative to each patient’s own baseline rather than as a single universal absolute threshold.

The clinical utility of PIP monitoring can be contextualized through Bayesian analysis (positive LR = 19.3; negative LR = 0.04) [23]: at a pre-test IAH probability of 16% [22], PPV reaches 78% and NPV approaches 99% [24]. Across prevalence scenarios of 5–30%, the near-perfect NPV is the most clinically actionable finding.

In clinical practice, PIP monitoring during ventral hernia repair functions primarily as an intraoperative “yellow flag” for IAP elevation. At critical procedural steps — namely, reduction of hernial contents and fascial closure — PIP provides a real-time, non-invasive estimate of IAP that requires no additional equipment or interruption of the operative field. Its greatest clinical value lies in its near-perfect negative predictive value: when PIP remains stable and does not rise significantly from baseline, there is a 99% probability that IAP has not reached the IAH threshold, effectively reassuring the surgical team that quaternary abdominal compartment syndrome is unlikely. Conversely, when PIP rises above 23 cmH₂O in patients with BMI < 25 kg/m² — or above 25 cmH₂O in those with BMI ≥ 25 kg/m² — the probability of concurrent IAH increases substantially, warranting prompt escalation to more intensive IAP monitoring, including formal bladder pressure measurement, and alerting the team to the risk of QACS. In this sense, PIP does not replace IAP monitoring; rather, it serves as a continuously available screening signal that guides when definitive measurement is indicated.

It is important to note that this study was not conducted in trauma patients or the intensive care unit. Previous work did not demonstrate IAP–AWP correlation in critically ill patients [22], which is expected given their fundamentally different cardiorespiratory dynamics. Our model is most applicable to the elective surgical setting.

### Limitations

This study has several limitations. First, findings are derived from a specific population — predominantly young, female, and overweight but non-obese patients undergoing elective laparoscopic cholecystectomy at a single center — and should not be extrapolated to critically ill, trauma, or intensive care settings. The study population consisted exclusively of ASA I–II patients; IAH risk may differ substantially in ASA III–IV patients with significant cardiorespiratory comorbidities, warranting separate investigation. Second, the smoker subgroup (*n* = 3) is too small for any definitive clinical inference. Third, IAP was measured via the insufflator readout rather than an independent calibrated pressure transducer; the insufflator display was not validated against a reference standard, which may introduce measurement uncertainty. Fourth, although BMI-stratified cutoffs are provided, external validation of both the 23 cmH₂O and 25 cmH₂O thresholds in complex hernia repair populations with loss-of-domain remains an essential next step.

## Conclusion

Peak inspiratory pressure is a reliable surrogate for intra-abdominal pressure during laparoscopic surgery and accurately detects intraoperative IAH. PLAT is not recommended as a standalone diagnostic surrogate for IAP, given its high ICC (0.936) indicating that most of its variability reflects fixed patient-level compliance rather than dynamic IAP changes. BMI-stratified optimal PIP cutoffs are 23 cmH₂O (BMI < 25) and 25 cmH₂O (BMI ≥ 25). Airway pressure monitoring may enhance intraoperative awareness of IAH/ACS and warrants prospective validation in overweight, obese, and complex hernia repair populations.
